# Investigations of a Possible Chemical Effect of* Salvadora persica *Chewing Sticks

**DOI:** 10.1155/2017/2576548

**Published:** 2017-04-18

**Authors:** Reham Albabtain, Muhammad Azeem, Zenebech Wondimu, Tulay Lindberg, Anna Karin Borg-Karlson, Anders Gustafsson

**Affiliations:** ^1^Department of Dental Medicine, Unit of Periodontology, Karolinska Institutet, P.O. Box 4064, 141 04 Stockholm, Sweden; ^2^Department of Dental Hygienist, Medical Sciences College, King Saud University, Riyadh, Saudi Arabia; ^3^KTH Royal Institute of Technology, School of Chemical Science and Engineering, Department of Chemistry, Ecological Chemistry Group, 100 44 Stockholm, Sweden; ^4^Department of Chemistry, COMSATS Institute of Information Technology, Abbottabad 22060, Pakistan

## Abstract

*Salvadora persica* is commonly used chewing sticks in many parts of the world as an oral hygiene tool. This study measured the amount of benzyl isothiocyanate (BITC) released into the mouth and assessed its retention time in saliva. The study also tested if the released amount of BITC could potentially be antibacterial or cytotoxic. Twelve subjects brushed their teeth with fresh Miswak once, twice, and four times. The amount of BITC in the saliva and in the used brushes was quantified using gas chromatography-mass spectrometry. The antibacterial effect of BITC and Miswak essential oil (MEO) was tested against* Haemophilus influenzae*,* Aggregatibacter actinomycetemcomitans,* and* Porphyromonas gingivalis*. The cytotoxic effect on gingival fibroblasts and keratinocytes was tested using MTT. The highest amount of the active compounds was detected in saliva after using the Miswak tip for once and immediately. It significantly decreased when the Miswak tip was used more than once and thus after 10 min. The growth of the tested bacteria was inhibited by MEO and BITC in a dose dependent manner,* P. gingivalis* being the most sensitive. MTT assay showed that BITC and MEO were cytotoxic towards gingival fibroblasts while oral keratinocytes showed resistance. This study suggests that the Miswak tip should be cut before each use to ensure the maximum effect.

## 1. Introduction

Humans have been interested in maintaining clean and healthy teeth since ancient time [1]. The use of various plants to maintain good oral hygiene and oral health is well-established in many parts of the world since time immemorial [1–3]. More than 180 plant species are known to be used for oral health; among them 150 are used in Africa [4]. Different parts of plants are used for this purpose but the twigs made from stems or roots are preferred [5].

The chewing sticks made from the roots of* Salvadora persica* are commonly used in major parts of Middle East, Africa, South Asia, and many parts of Europe and America [2, 5].* S. persica* is shrub like plant belonging to the family Salvadoraceae. It is widespread in large parts of Africa and Asia. In Middle East,* S. persica* or Arak tree is also known by several other names such as Siwak, Miswak, Mustard tree, and natural toothbrush tree. The use of chewing sticks (Miswak) made from* S. persica* is an old pre-Islamic custom adhered to by the ancient Arabs in order to make their teeth white and shiny [6]. When Islam was established in the Middle Eastern region and parts of Africa, it reinforced the use of Miswak as an important aspect of dental care. Today, the majority of Muslims prefer to use Miswak for oral hygiene in order to follow the Sunna of their Prophet [6]. In this study the word “Miswak” is equivalent to “chewing stick.”

A number of studies have shown that chewing sticks are effective in reducing plaque and gingival inflammation [7, 8]. When used properly, they could be as effective as a toothbrush [4, 7, 9–12] or even more [13]. The frequent use of chewing stick has been associated with a lower need for periodontal treatment [14]. However, some studies have reported more plaque formation and gingival bleeding in individuals who used chewing sticks compared to toothbrush [15–18]. The possibility of a chemical antibacterial effect adjunctive to a mechanical effect has been discussed in several publications. A chemical effect has been shown in vitro in a number of studies [5, 19, 20]. Previously, our research group has shown that both small pieces of* S. persica* root and essential oil exhibit a strong antibacterial effect, especially on Gram-negative bacteria [20–22]. Other studies showed a limited antibacterial effect of chewing gums and mouth rinses containing* S. persica* extract [23, 24].

There seems to be a discrepancy between a very clear antibacterial effect in vitro and a less obvious effect in vivo. Thus the aim of this study was to establish how much of the BITC is contained inside the fresh chewing stick that could be released into the mouth and how long it is retained in the saliva. Furthermore, we wanted to know if the released amount of BITC could be antibacterial and/or cell toxic.

## 2. Materials and Methods

### 2.1. Study Design

This study consists of three main parts, (i) measurements of the amounts of BITC released from the Miswak chewing sticks into the saliva during use, (ii) measurement of cytotoxicity, and (iii) measurement of antibacterial effect of Miswak oil and BITC.

### 2.2. Collection of Miswak Chewing Sticks

Fresh roots of* S. persica* were purchased directly from a farm in Jizan, Saudi Arabia and shipped to Karolinska Institutet, Stockholm, Sweden, by FedEx within 3 days after harvest. During the transportation, they were sealed by paper wrap and plastic bag as an outer layer. On reaching destination, they were manually cleaned, sorted, placed in airtight polyethylene bags, and stored at −80°C till used for essential oil extraction or using as chewing stick (Miswak) in different studies.

### 2.3. Extraction of Essential Oil

The essential oil was extracted by adopting methodology described by El-Seedi et al., 2012 [25]. Briefly, the fresh twigs of* S. persica* roots (1.4 kg) were cut into 20–30 mm long pieces and mixed with 700 mL double-distilled water. The resulting mixture was subjected to hydrodistillation for 4-5 h in a glass distillation apparatus. The collected distillate (oil/water mixture) was extracted three times with HPLC grade hexane (VWR International, Sweden) with the help of a separating funnel. Anhydrous MgSO_4_ (Alfa Aeser, UK) was added to the hexane extract to remove any trace of water. After filtration, hexane was evaporated using a rotary evaporator (Buchi Rotavapor R-210, Switzerland) at 20°C under reduced pressure. The essential oil obtained was then weighed and the yield calculated as percentage (w/w) of the total plant material. The essential oil was stored at −20°C in a freezer till further experiments and analyses on GC-MS.

### 2.4. Collection of Used Miswak Brushes and Saliva Samples

Twelve adults, five men and seven women, who reported good oral health were chosen for this study. The purpose of the study was fully explained and informed consent was obtained from all the participants before the study was commenced. The study was approved by the regional Research Ethics Committee in Stockholm (#2012/609-31/1). Each of the twelve participants was assigned with a fresh chewing twig of* S. persica *root that was 150 mm long and about 10 mm diameter. This twig was to be used as chewing stick throughout the entire study period. In order to expose fresh twig, each volunteer cut off roughly a 2 mm long piece from one side of the twig. From the remaining twig, approximately 7 mm long piece was cut; this piece was collected in a labelled Eppendorf tube so that the quantity of compounds originally present in each twig could be determined. After removal of the “baseline” piece, the 5–7 mm length of the twig was chewed and the resulting fibrous brush was used to clean the teeth. During the cleaning process saliva collected in mouth was transferred in a 15 mL Falcon tube. The used brush was then cut and placed in a labelled Eppendorf tube. The saliva and used Miswaks brushes were collected after the first, second, and fourth use of the twig. All the Eppendorf tubes and Falcon tubes were collected from the participants and stored in a freezer at −80°C pending extraction and analysis of volatile compounds using gas chromatography-mass spectrometry (GC-MS).

In order to check the retention of BITC and other compounds in saliva, the volunteers using freshly cut Miswak brush were also asked to collect saliva before brushing, immediately after brushing, and 5, 10, and 30 min after brushing. The saliva samples were treated as described above until analysed for the quantification of volatiles by GC-MS.

### 2.5. Collection of Volatiles from Saliva

The frozen saliva samples were thawed by being kept at room temperature for 1.5 h and then for another 30 min after covering with aluminum foil so that the headspace (HS) air in the tubes could be equilibrated with volatile compounds in saliva. The volatiles from the saliva samples were extracted by using HS solid phase microextraction (SPME) method [26–29]. After the equilibration of the HS the SPME syringe was immersed into the sampling tube through a pinhole in the aluminum foil covering the mouth of the tube and the SPME fibre was exposed to the HS of the saliva for 2 h for the collection of volatiles. The SPME fibre was retracted into the syringe and immediately injected into the gas chromatograph injector. The SPME consisted of 65 *μ*m stable flax fibre coated with polydimethylsiloxane/divinylbenzene (Supelco, USA). Prior to its first use the SPME fibre was conditioned at 250°C for 30 min as advised by the manufacturer.

### 2.6. Extraction of Chemicals from Fresh and Used Miswaks

The extraction of chemicals from the fresh and used Miswak brushes and the quantification was made by using previously reported method by Zhao et al., 2011 [30]. Pieces of chewing sticks, unused and used, were thawed for 1 hour at room temperature, cut into small pieces, and placed in preweighed and labelled glass vials. For chemical extraction, the twig pieces were dipped in 1 mL of n-hexane (HPLC grade) for 24 h at room temperature. The extracts were collected in fresh glass vials using a glass Pasteur pipette. The residual twig pieces were rinsed with another 500 *μ*L pure hexane and the extracts were pooled in the collection vials. Following the chemical extraction, the twig pieces were dried in an oven at 80°C for 15 h. Their dry mass was measured after keeping the oven dried pieces in desiccator to obtain constant mass. The dry mass of Miswak brushes was used to calculate the absolute amount of volatiles compounds present in the Miswak brushes. Hexane extract of twig pieces was injected (1 *μ*L) in GC-MS via liquid injection using a syringe.

### 2.7. Chemical Analysis of Saliva and Chewing Stick Pieces

Volatile compounds collected on a SPME fibre and extracted in hexane were separated and identified in GC-MS, using a Varian 3400 gas chromatograph (GC) (Varian, Palo Alto, CA, USA) connected to a Finnigan SSQ 7000 (Waltham, MA, USA) quadruple mass spectrometer with electron ionisation. The GC was equipped with a split/split less injector (split less mode, 30 sec); the carrier gas was helium (99.99%, Strandmöllen AB, Ljungby, Sweden), with a constant pressure of 10 psi. The GC was fitted with a DB-Wax capillary column (30 m, 0.25 mm ID, and 0.25 *μ*m film thickness, J & W Agilent, Santa Clara, CA, USA). The temperature of the GC oven was maintained at 45°C for 30 sec which was gradually raised up to 235°C at a rate of 8°C min^−1^ and maintained at 235°C for 5.75 min. The injector temperature was isothermally set at 230°C and the transfer line connecting the GC to the MS was isothermally set at 235°C. The MS ion source temperature was 150°C; mass spectra were obtained at 70 eV with a mass range from 30 m/z to 400 m/z in positive centroid mode. Separated compounds were initially identified by comparing their mass spectra to the NIST-08 (National Institute of Standard and Technology, USA) MS library. The final authentication was made by analysing pure standard compounds on GC-MS using the same parameters used for the chew twigs and saliva samples. Quantification of identified compounds was made by running different concentrations of standard compounds on GC-MS followed by producing standard curve.

## 3. Cytotoxicity

### 3.1. Cell Lines

Primary human gingival fibroblasts were established from gingival biopsies obtained from healthy patients with no clinical signs of periodontal disease. The protocol, including the collection of gingival biopsies, was approved by the Ethical Committee at Huddinge University Hospital (#377/98). Gingival fibroblasts were established and cultured in the manner previously described [31]. Gingival fibroblasts used for the experiments were cultured in 175 cm^2^ tissue culture flasks until reaching 70–80% confluence and the experiments were performed between 7th and 14th passage.

Oral keratinocytes (OKF6/TERT-2) [32] were cultured in EpiLife cell culture medium (Invitrogen, Carlsbad CA, USA) supplemented with 0.06 mM calcium, 10 *μ*g/mL gentamicin, 0.25 *μ*g/mL amphotericin B, and defined growth serum (EDGS) (Invitrogen, Carlsbad, CA, USA) in a humidified atmosphere of 5% CO_2_ at 37°C.

### 3.2. The Cell Viability Bioassay

The stock solutions (1 mg/mL) of* S. persica* roots essential oil (EO) or pure compounds BITC, benzaldehyde (BA), and benzyl cyanide (BC) were prepared in absolute ethanol. The solutions were sterilized by filtering them through a 0.22 *μ*m filter before the working concentrations of EO or pure compounds were made in Dulbecco's modified Eagle's medium (DMEM) by diluting the respective stock solutions.

The fibroblasts (1 × 10^4^) were seeded in 96-well plates in tetraplicate manner in DMEM supplemented with penicillin (50 U/mL), streptomycin (50 *μ*g/mL), and fetal calf serum (FCS) (5%) and cultured at 37°C for 24 h. The cell layers were rinsed twice with serum-free DMEM followed by the addition of 10 *μ*L serum-free medium containing different concentrations (0.4 *μ*g/mL–3 *μ*g/mL) of EO, BITC, BC, and BA. Control cells were treated with corresponding amounts of ethanol, added in serum-free DMEM to test the effect of ethanol on cells. After an incubation period of 24 h, the colorimetric assay, 3-(4,5-dimethylthiazol-2yl)-2,5-diphenyltetrazolium bromide (MTT) kit (Abnova, Taipei, Taiwan), was used to assess the viability of gingival fibroblast according to the manufacturer's instructions. Fibroblasts were incubated with MTT reagent for 4 h at 37°C and solubilizing solution was added. The resulting mixture was shaken for 1 h at room temperature and absorbance was measured at *λ*_max_ 507 nm on an ELISA reader.

Oral keratinocytes were seeded in 96-well plates until reaching 80% confluence; the medium was then removed and replaced with 100 *μ*L of medium with increasing concentrations of EO or BITC (5–100 *μ*g/mL) and incubated for 24 hrs. Cell viability was detected by adding MTT to the medium, incubating it for 4 hrs, and then adding DMSO to dissolve the formazan product. After the plate was shaken at low speed for 10 minutes absorbance was measured at 540 nm using an ELISA reader.

### 3.3. Effect of Exposure Time on Cell Viability

Human gingival fibroblast cells were seeded in a 96-well microtiter plate as previously mentioned, incubated for 24 h, washed with a serum-free medium, and then treated with EO, BITC, BC, or BA. The concentration of EO and BITC ranged from 1 *μ*g/mL to 100 *μ*g/mL, whereas for BC and BA it ranged from 0.5 *μ*g/mL to 20 *μ*g/mL. After an exposure time of 10 min, the cells were washed twice with a serum-free medium. The cells were then incubated for another 24 hrs after adding 100 *μ*L of complete medium. The cell-recovery assessment was carried out using the MTT kit described above. Each cell viability assay was performed using four replicates of all tested dilutions and the experiments were repeated three times.

## 4. Antibacterial Activity 

### 4.1. Bacterial Isolates and Growth Conditions

*S. persica* EO and its pure constituents were tested for antibacterial activity against 3 oral pathogenic bacteria:* Aggregatibacter actinomycetemcomitans* KH 1519,* Porphyromonas gingivalis*, ATCC 33277, and,* Haemophilus influenzae,* ATCC 49247 as a well characterized reference.* H. influenzae* was propagated for 16 h in chocolate agar plates in a 5% CO_2_ incubator.* A. actinomycetemcomitans* was propagated in blood agar plates for 40 h in 5% CO_2_ atmosphere.* P. gingivalis* was propagated for 136 h on Brucella agar plates supplemented with Hemin (0.05 mg/mL), vitamin K (0.01 mg/mL), and citrated horse blood (5%) in an anaerobic atmosphere created with gas-packs (GasPak™, Becton Dickinson, Franklin Lake, NJ, USA). All agar plates were prepared and retrieved from the substrate unit, at the laboratory of clinical microbiology, Karolinska Hospital, Huddinge, Sweden.

### 4.2. Antibacterial Dose-Response Assay

The antibacterial activity was performed by broth dilution assay according to previously described method [20, 33, 34] with modification. Freshly grown colonies of* H. influenza*,* A. actinomycetemcomitans,* and* P. gingivalis* were suspended in 2 mL of 0.9% NaCl solution and optical density (absorbance) of resultant suspension was measured at the wavelength of 590 nm by using a double beam spectrophotometer. The bacterial suspensions were then diluted to get optical density of 0.5; the number of colony forming units (CFU) was in the range of 10^7^–10^8^ per mL of suspension depending upon the bacteria. Prior to the antibacterial assay, the bacterial suspensions were further diluted 10,000 times in fresh saline media. The 495 *μ*L of the diluted bacterial suspension (10^3^–10^4^ CFU/mL) was poured in each Eppendorf tube to which 5 *μ*L of saline solution or dimethyl sulfoxide (DMSO) or test oil/compound solution was added. The test solutions of EO, BITC, BA, and BC were prepared by dissolving them in DMSO. The final concentration of the DMSO (solvent) was never more than 1% of the bacterial suspension in sample or control tubes. The final concentration of the test compounds or EO was in the range of 11.7 *μ*g/mL to 11700 *μ*g/mL of bacterial suspension and that of chlorhexidine was 500 *μ*g/mL. DMSO worked as a negative control whereas chlorhexidine was used as positive control for all tested bacteria. The contents of Eppendorf tubes were vortexed and incubated in a shaker for 10 min after that 100 *μ*L of suspension mixture from each Eppendorf tube was evenly spread on an agar plate with a respective growth medium. At least two replicates were run for each test or control sample and two replicates were plated by taking sample from each Eppendorf tube. The plates of* H. influenza* were incubated for 16 h,* A. actinomycetemcomitans* for 40 h, and* P. gingivalis* for 136 h. The bacterial colonies on each plate were counted by using a colony counter. The antibacterial bioassay of each test sample was repeated in triplicate manner.

### 4.3. Statistical Analysis

All data are presented as mean values and standard deviation of three separate experiments with two to three replicates. Two-tailed paired* t*-test was used to examine the significance differences between control and test samples. One way ANOVA with Bonferroni post hoc test was used to find difference between bacterial growth when they were treated with same concentration of Miswak essential oil or pure compounds. The statistical analyses were carried out using SPSS 20 computer software (IBM, USA).

## 5. Results

### 5.1. Chemical Analysis of* S. persica* Essential Oil

The hydrodistillation of* S. persica* roots twigs produced 1.4 g of essential oil giving rise 0.001% yield. GC-MS analysis of the oil revealed the presence of three major antibacterial compounds constituting 92% of the oil (Table 1). The main compounds in* S. persica* essential oil were benzyl isothiocyanate (BITC), (CID: 2346), 74.4% followed by benzyl cyanide (BC) (CID: 8794), 16.3%, and benzaldehyde (BA) (CID: 240), 0.7% (Table 1).

### 5.2. Quantitative Analysis of Miswak Pieces and Saliva

The amount of BITC found in unused Miswak pieces (base line) was statistically different (*p* < 0.05) from the amount of BITC found in Miswak brushes after one-, two-, and four-time use (Table 1). Moreover, the BITC amount in Miswak-1 brush was significantly more (*p* < 0.05) than Miswak-2 and Miswak-4 brushes; however, Miswak-2 and Miswak-4 exhibited similar amount (*p* > 0.05). The amount of benzyl cyanide decreased significantly after 2 and 4 uses (*p* < 0.05) while benzaldehyde contents remained the same (Table 1).

**Figure 1:**
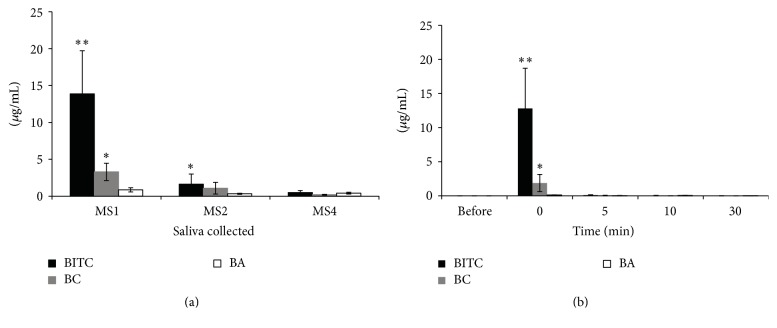
Benzyl isothiocyanate (BITC), benzyl cyanide (BC), and benzaldehyde (BA) concentration in saliva. (a) The concentration of compounds in saliva collected during fresh Miswak use (Saliva-1), saliva collected when Miswak was used for a second time (Saliva-2), and saliva collected while using Miswak for a fourth time (Saliva-4). (b) The concentration of compounds in saliva before brushing, immediately after brushing with fresh Miswak, and after 5, 10, and 30 min of Miswak brushing. In the above figure the bars denoted with *∗* and *∗∗* are significantly different when the comparison was made between the concentration of same compound detected in different samples.

The mean concentration of BITC in saliva samples after one-time use of the Miswak brush was 13.85 *μ*g/mL whereas the amount decreased drastically to 1.65 *μ*g/mL and 0.5 *μ*g/mL after the same Miswak brush was used two times and four times, respectively (Figure 1(a)). BITC and benzyl cyanide contents in saliva were statically different (*p* < 0.05) when fresh Miswak was used to clean the teeth (Figure 1(a)). When the previously used Miswak was used the amount of BITC and BC was very low compared to saliva-1.

### 5.3. Salivary Retention after Brushing with* S. persica* Miswak

The concentration of BITC in saliva immediately after using fresh Miswak was 13.8 *μ*g/mL. However, there was a marked reduction over time in the amount of all compounds (BITC, BC, and BH) found in the saliva, and it disappeared completely after 10 min (Figure 1(b)).

## 6. Cytotoxicity 

### 6.1. Gingival Fibroblasts

**Figure 2:**
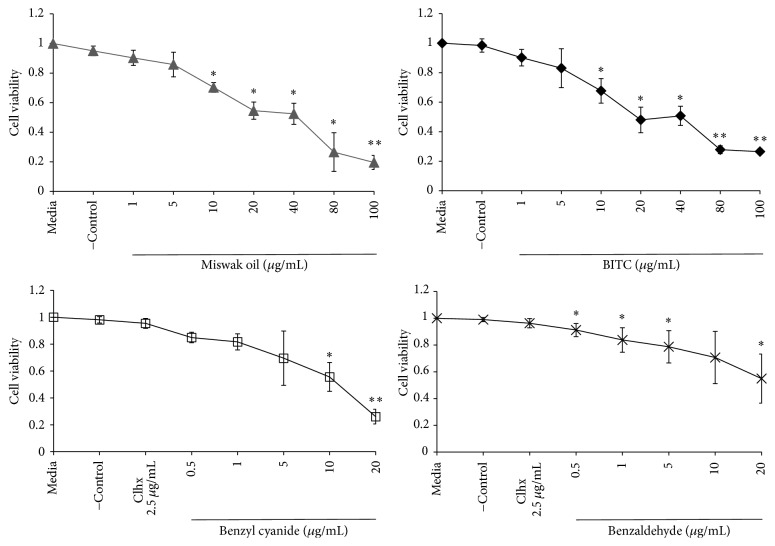
Gingival fibroblasts cells exposed for 10 min to different concentrations of Miswak oil, benzyl isothiocyanate (BITC), benzyl cyanide, benzaldehyde, and 2.5 *μ*g/mL of chlorhexidine. The bars denoted with *∗* (*p* < 0.05), *∗∗* (*p* < 0.01) are significantly different from negative control (DMSO) and media control.

A 10 min exposure to 1–5 *μ*g/mL of Miswak oil or BITC did not affect the cell viability of gingival fibroblasts compared to the negative control. A significant decrease in cell viability was observed at concentration of 10 *μ*g/mL or higher (*p* < 0.05) (Figure 2). The cells exposed to 2.5 *μ*g/mL chlorhexidine showed similar viability as control cells (*p* > 0.05). The most cytotoxic compound was benzaldehyde that decreased the cell viability even at lower concentration 0.5 *μ*g/mL (Figure 2). However, there was no significant effect of a 10 min exposure to 0.5–5 *μ*g/mL of benzyl cyanide except for 10–20 *μ*g/mL (*p* < 0.05) (Figure 2).

**Figure 3:**
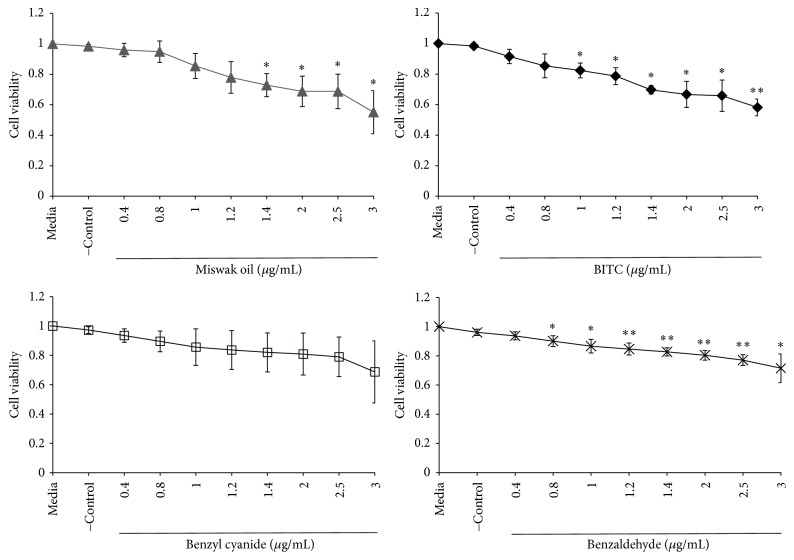
Gingival fibroblasts cell exposed for 24 h to different concentrations of miswak essential oil, benzyl isothiocyanate (BITC), benzyl cyanide, and benzaldehyde. The bars denoted with *∗* (*p* < 0.05), *∗∗* (*p* < 0.01) are significantly different from negative control (DMSO) and media control.

A significant decrease of cell viability was noted after a 24-hour exposure to Miswak oil at concentration 1.4 *μ*g/mL or higher (*p* < 0.05) and to BITC at concentrations ≥1 *μ*g/mL (*p* < 0.05) (Figure 3). Twenty-four-hour exposure to benzyl cyanide (0.4–3 *μ*g/mL) showed no significant effect on cell viability whereas benzaldehyde showed significant cell toxicity at concentration of 0.8 *μ*g/mL or higher (Figure 3).

### 6.2. Oral Keratinocytes

**Figure 4:**
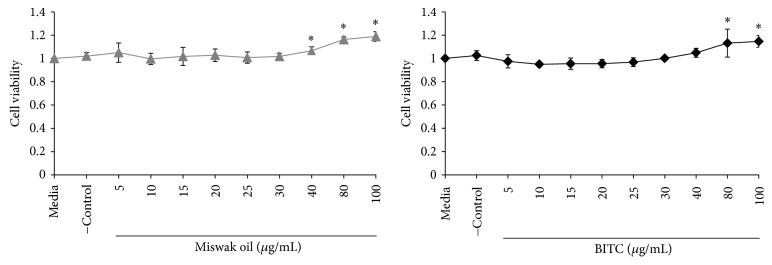
Oral keratinocytes exposed for 24 hr to different concentrations of Miswak oil and benzyl isothiocyanate (BITC). The points indicate cell viability in relation to the viability using media alone. Points marked with *∗* indicate a statistical difference from media control (*p* < 0.05).

The Miswak oil and BITC did not show any bad effect on the viability of keratinocytes when they were stimulated with Miswak oil or BITC for 24 h at the concentrations 5–100 *μ*g/mL; however, at higher concentration they show better cell viability (Figure 4) than control (*p* < 0.05).

### 6.3. Antimicrobial Effect of Miswak Essential Oil and Pure Compounds

**Figure 5:**
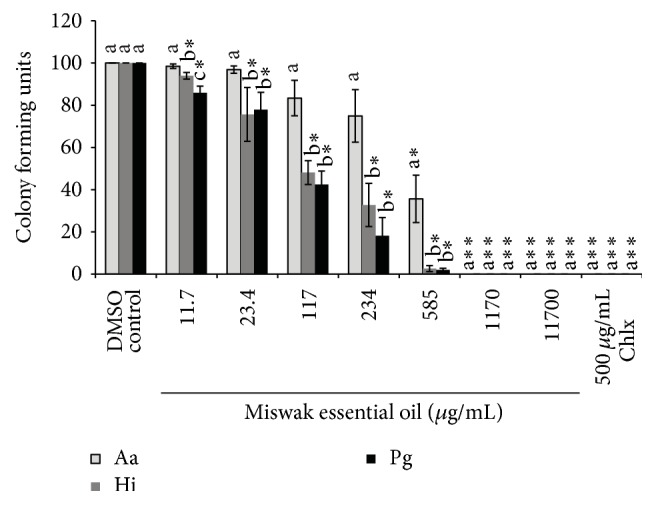
Inhibition of growth of* A. actinomycetemcomitans* (Aa),* H. influenzae* (Hi), and* P. gingivalis* (Pg). The individual bars represent the number of colony forming units (CFU) at various concentrations of Miswak oil or chlorhexidine (Chlx) compared to the negative control (DMSO). In the above figure *∗* stands for *p* < 0.01–0.001 and *∗∗* stands for *p* < 0.0001 when number of CFU were compared between negative control and different concentrations of Miswak essential oil or Chlx. Different letters on the bars represent significant difference (*p* < 0.05) between the CFU of the three bacteria tested at a given concentration of Miswak essential oil or Chlx independently, b indicate a significant difference compared to a, and c indicate a difference compared to both a and b.

**Figure 6:**
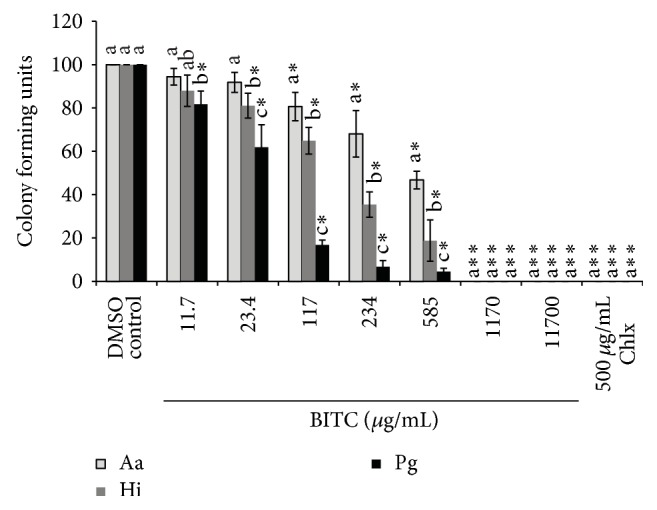
Inhibition of growth of* A. actinomycetemcomitans* (Aa),* H. influenzae* (Hi), and* P. gingivalis* (Pg). The individual bars represent the number of colony forming units (CFU) at various concentrations of benzyl isothiocyanate (BITC) or chlorhexidine (Chlx) compared to the negative control (DMSO). In the above figure *∗* stands for *p* < 0.01–0.001 and *∗∗* stands for *p* < 0.0001 when number of CFU were compared between negative control and different concentrations of BITC or Chlx. Different letters on the bars represent significant difference (*p* < 0.05) between the CFU of the three bacteria tested at a given concentration of BITC or Chlx independently, b indicate a significant difference compared to a, and c indicate a difference compared to both a and b.

Miswak essential oil and BITC showed a dose-dependent antibacterial activity against the three bacteria tested (Figures 5 and 6).* H. influenzae* and* P. gingivalis *were more susceptible towards Miswak essential oil; thus their growth was significantly inhibited (*p* < 0.01) at lowest concentration (11.7 *μ*g/mL) tested in experiments (Figure 5). In the presence of Miswak essential oil the growth of three tested bacteria was significantly different (*p* < 0.05) from each other (Figure 5). In contrast to Miswak oil the BITC only inhibited the growth of* P. gingivalis* at lowest concentration (Figure 6). Chlorhexidine completely inhibited the growth of all tested bacterial species at a concentration 500 *μ*g/mL (Figures 5 and 6).

**Figure 7:**
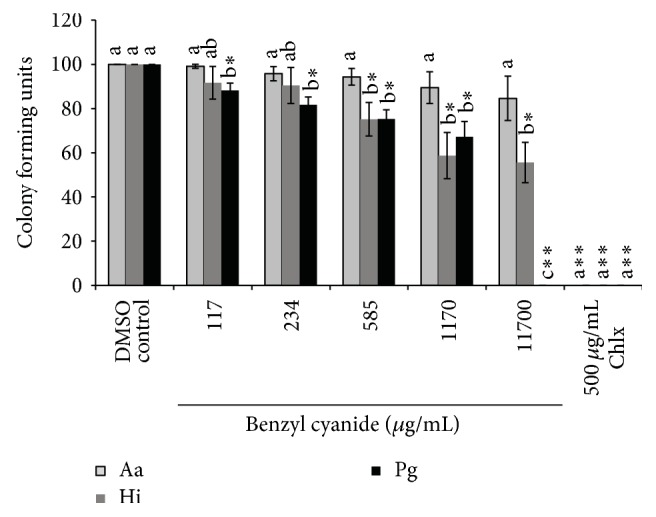
Inhibition of growth of* A. actinomycetemcomitans* (Aa),* H. influenzae* (Hi), and* P. gingivalis* (Pg). The individual bars represent the number of colony forming units (CFU) at various concentrations of benzyl cyanide or chlorhexidine (Chlx) compared to the negative control (DMSO). In the above figure *∗* stands for *p* < 0.01–0.001 and *∗∗* stands for *p* < 0.0001 when number of CFU were compared between negative control and different concentrations of benzyl cyanide or Chlx. Different letters on the bars represent significant difference (*p* < 0.05) between the CFU of the three bacteria tested at a given concentration of benzyl cyanide or Chlx independently, b indicate a significant difference compared to a, and c indicate a difference compared to both a and b.

**Figure 8:**
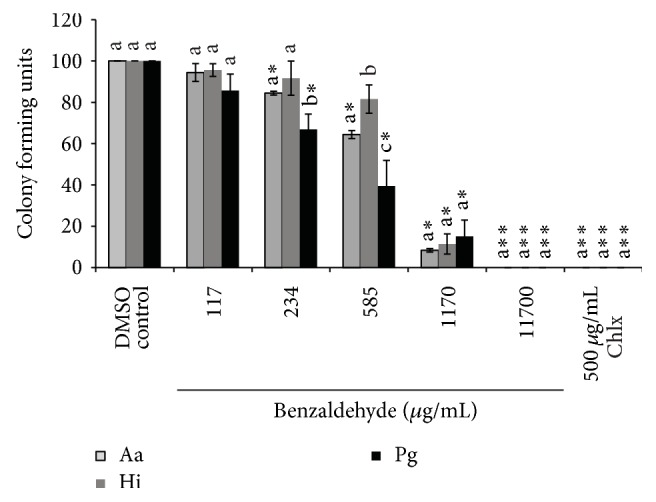
Inhibition of growth of* A. actinomycetemcomitans* (Aa),* H. influenzae* (Hi), and* P. gingivalis* (Pg). The individual bars represent the number of colony forming units (CFU) at various concentrations of benzaldehyde or chlorhexidine (Chlx) compared to the negative control (DMSO), set to 100%. In the above figure *∗* stands for *p* < 0.01–0.001 and *∗∗* stands for *p* < 0.0001 when number of CFU were compared between negative control and different concentrations of benzaldehyde or Chlx. Different letters on the bars represent significant difference (*p* < 0.05) between the CFU of the three bacteria tested at a given concentration of benzaldehyde or Chlx independently, b indicate a significant difference compared to a, and c indicate a difference compared to both a and b.

Benzaldehyde showed better activity than benzyl cyanide against bacterial strains; however, both compounds were not able to inhibit the bacterial growth at concentration found in saliva or Miswak brushes (Figures 7 and 8).

## 7. Discussion

The present study shows that the main antibacterial compound, BICT, released from Miswak chewing sticks into the saliva. However, the study also shows that the released BITC was only retained in the saliva for a very short time. This is most likely due to a combination of the volatile nature of BITC [35] and the rinsing effect of the saliva. Earlier in vitro studies demonstrated a strong antibacterial effect, especially on Gram-negative bacteria associated with periodontal disease [20, 22]. However, our group failed to clinically show a chemical effect [36]. In order to clinically demonstrate a chemical effect, it is possible that a more frequent use of Miswak more than twice daily would have been required. Previously, Gazi et al. 1990 [11] reported significantly better results when Miswak was used five times a day than when it was used twice daily in subjects with a high standard of oral hygiene. It is also possible that it takes longer than the three weeks used in our previous studies [36] for a weak antibacterial effect to show a clinical effect. Earlier, many studies have shown a beneficial effect of Miswak compared to tooth brushing when the Miswak was used regularly [14, 37, 38].

We are also able to show that the amount of BITC released from the Miswak reduced drastically if the same Miswak is used more than once. Taken together, these findings suggest that in order to achieve any chemical effect besides the mechanical it would be necessary to use a fresh piece of Miswak each time. This finding is valuable information for the large number of habitual Miswak users. Many studies have advocated the use of Miswak, but no previous study recommended users to regularly change/cut the tip of the Miswak in order to optimize its effect.

Our antibacterial tests showed that* P. gingivalis *was the most sensitive of the three tested bacteria. This is particularly interesting since this bacterium is considered a keystone pathogen in the etiology of periodontitis [39]. The other possible antibacterial compounds of Miswak, benzaldehyde and benzyl cyanide, showed less inhibitory effects on the tested bacteria.

The cytotoxic effects of Miswak oil and BITC were highly dependent on exposure time. After 10 minutes of exposure to Miswak oil and BITC the gingival fibroblasts viability was significantly reduced at concentrations of 10 *μ*g/mL and above, which is below the average concentration detected in saliva immediately after brushing with fresh Miswak brush. Increasing the exposure time to 24 h significantly affected the viability of gingival fibroblasts at concentrations from 1.4 *μ*g/mL. Cultivated fibroblasts are very sensitive to cytotoxicity assays and the results cannot be compared to an in vivo situation in the mouth.

The very high sensitivity of cultivated fibroblasts towards chlorhexidine is well illustrated in previous studies where exposing these cells to low concentrations of chlorhexidine (0.00250.12%) showed 100% mortality of the cells [40, 41]. This is also in agreement with results of our group showing that 0.05% of chlorhexidine kills the gingival fibroblast cells within seconds (Albabtain et al. unpublished data). This concentration is much lower than the recommended mouthwash concentration. In a previous study, ethanol extract of* S. persica* was found to be nontoxic to human gingival fibroblasts when using three different cytotoxic assays at 1 mg/mL concentration [42].

The oral keratinocytes proved to be a much more resistant cell type. There was no decrease in cell viability when the keratinocytes were exposed to the same concentrations as the gingival fibroblasts. These results are in agreement with a previous study where oral keratinocytes were found more resistant towards long-chain bases compared with oral fibroblasts [43]. Moreover, the oral mucosa is much more resistant to toxic substances than a monolayer cultured cells, because of the mucin and the keratin layers present in it [44]. Therefore, the Miswak constituent (BITC) could be well tolerated in vivo. An early study by Ezmirly et al. [45] reported that neither aqueous nor ethanolic* S. persica* Miswak extract was toxic to mice at doses of up to 1200 mg/kg.

## 8. Conclusion

This study shows that BITC is released from the Miswak into the oral cavity but that retention time is very short. The amount of BITC decreases gradually if the same piece of Miswak is used several times. These observations strongly suggest that the Miswak should be cut before each use to ensure that fresh, unexposed Miswak is used to get the maximum effect.

## Figures and Tables

**Table 1:** Chemical composition of *S. persica* roots essential oil and the amount of benzyl isothiocyanate, benzyl cyanide, and benzaldehyde in Miswak brushes.

Sample	Benzyl isothiocyanate	Benzyl cyanide	Benzaldehyde
Essential oil	74.42%	16.26%	0.67%

Amount of compounds in Miswak brushes (mg/g dry wt)			

Miswak-0	6.49 ± 1.14^a^	0.190 ± 0.036^a^	0.005 ± 0.001^a^
Miswak-1	3.52 ± 0.66^b^	0.129 ± 0.034^b^	0.006 ± 0.002^a^
Miswak-2	2.01 ± 0.42^c^	0.072 ± 0.016^c^	0.007 ± 0.002^a^
Miswak-4	1.49 ± 0.51^c^	0.037 ± 0.012^d^	0.005 ± 0.001^a^

Miswak-0 stands for baseline piece, that is, the amount of compounds present originally in the Miswak pieces. Miswak-1 stands for amount of compounds found in miswak brushes after one-time use; similarly Miswak-2 and Miswak-4 stand for amount of compounds in Miswak brushes after two- and four-time use. Different letters on values are indicating statistical difference (*p* < 0.05) between values presented in the same column. Each value expressed in the table is a mean ± standard error (*N* = 12).

## References

[1] Wu C. D., Darout I. A., Skaug N. (2001). Chewing sticks: timeless natural toothbrushes for oral cleansing. *Journal of Periodontal Research*.

[2] Al lafi T., Ababneh H. (1995). The effect of the extract of the miswak (chewing sticks) used in Jordan and the Middle East on oral bacteria. *International Dental Journal*.

[3] More G., Tshikalange T. E., Lall N., Botha F., Meyer J. J. M. (2008). Antimicrobial activity of medicinal plants against oral microorganisms. *Journal of Ethnopharmacology*.

[4] Elvin-Lewis M. (1980). Plants used for tooth cleaning throughout the world. *Journal of Preventive Dentistry*.

[5] Almas K. (1999). The antimicrobial effects of extracts of Azadirachta indica (Neem) and Salvadora persica (Arak) chewing sticks. *Indian Journal of Dental Research*.

[6] Bos G. (1993). The miswāk, an aspect of dental care in islam. *Medical History*.

[7] Olsson B. (1978). Efficiency of traditional chewing sticks in oral hygiene programs among Ethiopian schoolchildren. *Community Dentistry and Oral Epidemiology*.

[8] Al-Otaibi M., Al-Harthy M., Söder B., Gustafsson A., Angmar-Månsson B. (2003). Comparative effect of chewing sticks and toothbrushing on plaque removal and gingival health. *Oral Health & Preventive Dentistry*.

[9] Manley J. L., Limongelli W. A., Williams A. C. (1975). The chewing stick: its uses and relationship to oral health. *The Journal of Preventive Dentistry*.

[10] Nörmark S., Mosha H. J. (1989). Relationship between habits and dental health among rural Tanzanian children. *Community Dentistry and Oral Epidemiology*.

[11] Gazi M., Saini T., Ashri N., Lambourne A. (1990). Meswak chewing stick versus conventional toothbrush as an oral hygiene aid. *Clinical preventive dentistry*.

[12] Darout I. A., Albandar J. M., Skaug N. (2000). Periodontal status of adult Sudanese habitual users of miswak chewing sticks or toothbrushes. *Acta Odontologica Scandinavica*.

[13] Malik A. S., Shaukat M. S., Qureshi A. A., Abdur R. (2014). Comparative effectiveness of chewing stick and toothbrush: a randomized clinical trial. *North American Journal of Medical Sciences*.

[14] Al-Khateeb T. L., O'Mullane D. M., Whelton H., Sulaiman M. I. (1991). Periodontal treatment needs among Saudi Arabian adults and their relationship to the use of the Miswak. *Community Dental Health*.

[15] Eid M. A., al-Shammery A. R., Selim H. A. (1990). The relationship between chewing sticks (Miswak) and periodontal health. 2. Relationship to plaque, gingivitis, pocket depth, and attachment loss. *Quintessence International*.

[16] Mumghamba E. G., Markkanen H. A., Honkala E. (1995). Risk factors for periodontal diseases in Ilala, Tanzania. *Journal of Clinical Periodontology*.

[17] Mengel R., Eigenbrodt M., Schünemann T., Florès‐de‐Jacoby L. (1996). Periodontal status of a subject sample of Yemen. *Journal of Clinical Periodontology*.

[18] Norton M. R., Addy M. (1989). Chewing sticks versus toothbrushes in West Africa. A pilot study. *Clinical Preventive Dentistry*.

[19] Al-Bagieh N. H., Idowu A., Salako N. O. (1994). Effect of aqueous extract of miswak on the in vitro growth of Candida albicans. *Microbios*.

[20] Sofrata A., Santangelo E. M., Azeem M., Borg-Karlson A.-K., Gustafsson A., Pütsep K. (2011). Benzyl isothiocyanate, a major component from the roots of Salvadora persica is highly active against Gram-Negative bacteria. *PLoS ONE*.

[21] Sofrata A. H. (2010). *Salvadora Persica (MISWAK): An Effective Way of Killing Oral Pathogens*.

[22] Sofrata A. H., Claesson R. L. K., Lingström P. K., Gustafsson A. K. (2008). Strong antibacterial effect of miswak against oral microorganisms associated with periodontitis and caries. *Journal of Periodontology*.

[23] Amoian B., Moghadamnia A. A., Barzi S., Sheykholeslami S., Rangiani A. (2010). Salvadora Persica extract chewing gum and gingival health: improvement of gingival and probe-bleeding index. *Complementary Therapies in Clinical Practice*.

[24] Khalessi A. M., Pack A. R. C., Thomson W. M., Tompkins G. R. (2004). An in vivo study of the plaque control efficacy of Persica™: a commercially available herbal mouthwash containing extracts of *Salvadora persica*. *International Dental Journal*.

[25] El-Seedi H. R., Khalil N. S., Azeem M. (2012). Chemical composition and repellency of essential oils from four medicinal plants against ixodes ricinus nymphs (Acari: Ixodidae). *Journal of Medical Entomology*.

[26] Azeem M., Rajarao G. K., Nordenhem H., Nordlander G., Borg-Karlson A. K. (2013). *Penicillium expansum* volatiles reduce pine weevil attraction to host plants. *Journal of Chemical Ecology*.

[27] Azeem M., Rajarao G. K., Terenius O. (2015). A fungal metabolite masks the host plant odor for the pine weevil (*Hylobius abietis*). *Fungal Ecology*.

[28] Azeem M., Terenius O., Rajarao G. K. (2015). Chemodiversity and biodiversity of fungi associated with the pine weevil Hylobius abietis. *Fungal Biology*.

[29] Shah R. M., Azhar F., Shad S. A., Walker W. B., Azeem M., Binyameen M. (2016). Effects of different animal manures on attraction and reproductive behaviors of common house fly, Musca domestica L. *Parasitology Research*.

[30] Zhao T., Krokene P., Hu J. (2011). Induced terpene accumulation in Norway spruce inhibits bark beetle colonization in a dose-dependent manner. *PLoS ONE*.

[31] Yucel-Lindberg T., Olsson T., Kawakami T. (2006). Signal pathways involved in the regulation of prostaglandin E synthase-1 in human gingival fibroblasts. *Cellular Signalling*.

[32] Dickson M. A., Hahn W. C., Ino Y. (2000). Human keratinocytes that express hTERT and also bypass a p16(INK4a)- enforced mechanism that limits life span become immortal yet retain normal growth and differentiation characteristics. *Molecular and Cellular Biology*.

[33] Wiegand I., Hilpert K., Hancock R. E. W. (2008). Agar and broth dilution methods to determine the minimal inhibitory concentration (MIC) of antimicrobial substances. *Nature Protocols*.

[34] Ocheng F., Bwanga F., Joloba M. (2015). Essential oils from ugandan aromatic medicinal plants: chemical composition and growth inhibitory effects on oral pathogens. *Evidence-based Complementary and Alternative Medicine*.

[35] Tang C.-S., Takenaka T. (1983). Quantitation of a bioactive metabolite in undisturbed rhizosphere—Benzyl isothiocyanate from Carica papaya L.. *Journal of Chemical Ecology*.

[36] Sofrata A., Brito F., Al-Otaibi M., Gustafsson A. (2011). Short term clinical effect of active and inactive Salvadora persica miswak on dental plaque and gingivitis. *Journal of Ethnopharmacology*.

[37] Darout I. A., Albandar J. M., Skaug N., Ali R. W. (2002). Salivary microbiota levels in relation to periodontal status, experience of caries and miswak use in Sudanese adults. *Journal of Clinical Periodontology*.

[38] Al-Otaibi M., Al-Harthy M., Gustafsson A., Johansson A., Claesson R., Angmar-Månsson B. (2004). Subgingival plaque microbiota in Saudi Arabians after use of miswak chewing stick and toothbrush. *Journal of Clinical Periodontology*.

[39] How K. Y., Song K. P., Chan K. G. (2016). Porphyromonas gingivalis: an overview of periodontopathic pathogen below the gum line. *Frontiers in Microbiology*.

[40] Eick S., Goltz S., Nietzsche S., Jentsch H., Pfister W. (2011). Efficacy of chlorhexidine digluconate-containing formulations and other mouthrinses against periodontopathogenic microorganisms. *Quintessence International*.

[41] Schmidt J., Zyba V., Jung K. (2016). Cytotoxic effects of octenidine mouth rinse on human fibroblasts and epithelial cells—an in vitro study. *Drug and Chemical Toxicology*.

[42] Balto H. A., Al-Manei K. K., Bin-Mohareb T. M., Shakoor Z. A., Al-Hadlaq S. M. (2014). Cytotoxic effect of Salvadora persica extracts on human gingival fibroblast cells. *Saudi Medical Journal*.

[43] Poulsen C., Mehalick L. A., Fischer C. L. (2015). Differential cytotoxicity of long-chain bases for human oral gingival epithelial keratinocytes, oral fibroblasts, and dendritic cells. *Toxicology Letters*.

[44] Dahl J. E., Frangou-Polyzois M. J., Polyzois G. L. (2006). In vitro biocompatibility of denture relining materials. *Gerodontology*.

[45] Ezmirly S. T., Cheng J. C., Wilson S. R. (1979). Saudi Arabian medicinal plants: Salvadora persica. *Planta Medica*.

